# Effectiveness of acupuncture for equine laminitis: Systematic review and meta-analysis

**DOI:** 10.14202/vetworld.2025.60-66

**Published:** 2025-01-09

**Authors:** Faisal Fikri, Agus Purnomo, Salipudin Tasil Maslamama, Muhammad Thohawi Elziyad Purnama

**Affiliations:** 1 Division of Veterinary Medicine, Department of Health and Life Sciences, Faculty of Health, Medicine and Life Sciences, Universitas Airlangga, Banyuwangi, East Java, 68425, Indonesia; 2 Research Group of Animal Biomedical and Conservation, Faculty of Health, Medicine, and Life Sciences, Universitas Airlangga, Banyuwangi, East Java, 68425, Indonesia; 3 Department of Veterinary Surgery and Radiology, Faculty of Veterinary Medicine, Universitas Gadjah Mada, Yogyakarta, 55281, Indonesia; 4 Department of Agricultural Biotechnology, Faculty of Agriculture, Eskisehir Osmangazi University, Eskisehir, 26040, Türkiye; 5 Department of Biology, Graduate School of Natural and Applied Sciences, Eskisehir Osmangazi University, Eskisehir, 26040, Türkiye

**Keywords:** acupuncture, domesticated animals, equine laminitis, horse, meta-analysis

## Abstract

**Background and Aim:**

In the past 20 years, acupuncture has been utilized as an alternative therapy for equine laminitis despite a lack of clinical evidence to support its effectiveness. Information from previous studies needs to be evaluated holistically to verify the effectiveness of acupuncture. This meta-analysis aimed to comprehensively investigate the effectiveness of acupuncture as a treatment for laminitis in horses.

**Materials and Methods:**

A total of 7 studies out of 145 were selected in the PubMed, Scopus, Cochrane Library, and ProQuest databases using the keywords “equine laminitis,” “acupuncture,” “horses,” and “lameness score.” Articles were selected following the Preferred Reporting Items for Systematic Reviews and Meta-Analysis flow diagram, and the extracted data were analyzed using OpenMEE software to determine Hedges’ d effect size and Log Odds Ratio.

**Results:**

As a result, this meta-analysis study reported that acupuncture improves horses with laminitis (Odds Ratio = 2.254; 95% CI = 1.167–4.355) and has a favorable effect on lameness scores (mean difference = −5.008; 95% CI = −8.094–−1.923).

**Conclusion:**

This meta-analysis enhanced the clinical studies demonstrating that twice-weekly acupuncture performed for 4 weeks consecutively can ameliorate lameness scores and a horse’s potential for recovery. These investigations have led to the implementation of dry needling, hemo-acupuncture, aqua-acupuncture, and electroacupuncture as alternate treatments for equine laminitis.

## INTRODUCTION

Laminitis was identified as an illness as early as 380 BCE by the Xenophon of Athens [1]. Laminitis is a complex and interconnected accumulation of vascular and inflammatory responses that affect hoof lamellar tissues. These episodes lead to an imbalance in interdigitation between the dermal and epidermal lamellae, causing considerable discomfort and incapacity in the equine population [2]. It is characterized by lesions involving the intricate system of interdigitated keratinized lamellae, which maintain a strong connection between the third phalanx bone and the epidermal hoof wall. This interdigitation may fail, and the underpinning third phalanx may move away from the wall because of damage to the lamellae [3]. According to studies conducted in Finland, 90% of horses initially appearing with lameness have endocrine-related laminitis, also known as endocrinopathic laminitis [4]. A previous study of risk factors for lameness in horses in the UK revealed that horses aged 6–9 and 10–15 years had a statistically significantly higher risk of laminitis than horses aged under 6 years. In addition, jumping horses had a higher risk than riding horses. Based on breed, Thoroughbreds had a higher risk than Warmbloods. Meanwhile, horses with BW: high ratios in the upper two quartiles (3.45–3.71 and >3.71, respectively) had a higher risk than the lowest quartile (<3.19) [5]. Because of irreversible injury to the hoof, affected horses may experience recurring episodes of injury and occasionally require euthanasia. It is unlikely that the foundered foot recovers to its original state after the fatal pathological cascade of laminitis has commenced because of severe anatomical dislocations [6].

In several previously reported clinical studies, allopathic treatments have not been successful in treating laminitis. However, despite the paucity of evidence-based scientific studies, there is indirect evidence that acupuncture improves lameness and pain in horses [7–13]. Traditional Chinese medicine includes acupuncture, extensively used to promote human and animal healing. The use of contemporary research instruments, such as sophisticated imaging techniques, reveals that acupuncture initiates a cascade of reactions associated with the release of endogenous opioid-like substances, such as enkephalins, endorphins, and endomorphins, which may be present in plasma and cerebrospinal fluids. However, its exact mechanism of action remains unknown. It has also been demonstrated that the limbic system is crucial for analgesia induced by acupuncture [14].

The application of acupuncture in equine medicine has advanced over the past few decades; however, few controlled clinical studies have investigated its efficacy. Anecdotal evidence suggests that acupuncture can effectively treat a range of excruciating orthopedic diseases, particularly back pain [15]. The question of whether acupuncture benefits horses, however, still needs to be answered due to the shortage of controlled trials with objective evaluations. It was essential to thoroughly review the effectiveness of acupuncture for laminitis in horses based on the lameness score and number of recovered horses in this meta-analysis.

## MATERIALS AND METHODS

### Ethical approval

Ethical clearance was not required because this study did not involve any animals. We used Preferred Reporting Items for Systematic Reviews and Meta-Analysis (PRISMA) guidelines for this meta-analysis.

### Study period and location

The screening procedure for relevant literature, a compilation of data, and analysis of data was performed from March to June 2024 at the Department of Biology, Faculty of Science, Eskisehir Osmangazi University, Eskisehir, Türkiye.

### Search strategy and study selection

A systematic screening process of the PubMed, Scopus, Cochrane Library, and ProQuest databases was used to identify relevant studies addressing the efficacy of acupuncture, laser puncture, and electroacupuncture during equine laminitis treatment. The study questions (P, population = equine laminitis; I, intervention = acupuncture; C, comparison = control; and O, outcomes = lameness scores or lameness locator) were developed using the PICO algorithm (Table 1). The search phrases “equine laminitis, acupuncture, lameness score” were the most pertinent. The Medical Subject Headings (MeSH) term’s inclusion of all pertinent and thorough keywords was confirmed. The database sample search algorithm was as follows: #1 “equine laminitis”[MeSH Terms] OR “horses”[All Fields] OR “horse”[All Fields]) AND “laminitis”[Title/Abstract]) #2 “acupuncture”[MeSH Terms] OR “acupuncture therapy”[Title/Abstract] OR “laserpuncture”[Title/Abstract] OR “acupuncture point”[Title/Abstract] OR “acupuncture points”[Title/Abstract] #3 “laminitis”[MeSH Terms] AND “score”[MeSH Terms] OR “lameness score”[Title/Abstract]).

**Table 1:** Searching strategy based on PICO methods.

PICO items	PICO	Keywords
Problems, Patients, and Populations	Equine laminitis	“Equine laminitis”[MeSH Terms] OR “horses”[All Fields] OR “horse”[All Fields]) AND “laminitis”[Title/Abstract])
Intervention	Acupuncture	“Acupuncture”[MeSH Terms] OR “acupuncture therapy”[Title/Abstract] OR “laserpuncture”[Title/Abstract] OR “acupuncture point”[Title/Abstract] OR “acupuncture points”[Title/Abstract]
Comparison, control	Control	“Control groups”[MeSH Terms]
Outcomes	Primary outcome: lameness score Secondary Outcomes: Lameness Level, Lameness Locator	“Laminitis”[MeSH Terms] AND “score”[MeSH Terms] OR “lameness score”[Title/Abstract])

### Eligibility criteria

**Figure 1:**
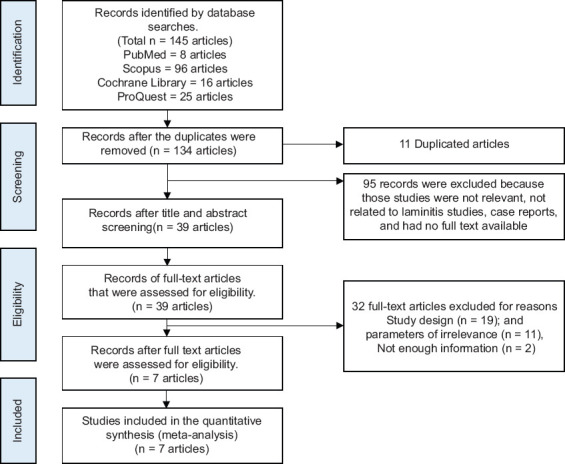
PRISMA flow diagram of the study selection process.

We used the PRISMA flow chart (Figure 1) to retrieve the relevant studies with the following inclusion and exclusion criteria.

### Inclusion criteria

Original research articles in English, available in full text and open access, reporting *in vivo* studies and randomized clinical trials, applying acupuncture for equine laminitis, and determining the lameness score.

### Exclusion criteria

Duplicate studies from the database, applications of acupuncture other than for laminitis, irrelevant studies, articles not in English, full text not available, case reports, and literature reviews.

### Data extraction

The information revealed based on the data characteristics was classified as follows: Study references, country, study period, horse breed, acupuncture method, acupuncture point, and therapy period. Data extracted for quantitative analysis included the total number of samples, number of recovered horses, and lameness score.

### Statistical analysis

Statistical analysis was performed using OpenMEE software [16]. The extracted data were then tabulated and entered with the “Continuous” category for the lameness scores and the “Count” category for the number of samples and recovered horses. A standard meta-analysis was used to evaluate the lameness scores of the recovered horses. Lameness scores are represented as mean difference ± standard deviation by reflecting Hedges’ d effect size and variance. Meanwhile, the number of recovered horses was represented by the log odds ratio. Heterogeneity of data was considered if p < 0.05 and I^2^ value > 50%. The output of the analysis was represented by forest and funnel plots to evaluate the distribution of data bias.

## RESULTS

### Identification and study selection

A total of 145 articles were identified from five electronic databases (PubMed found 8 articles, Scopus 96 articles, Cochrane Library 16 articles, and ProQuest 25 articles). Of these, 95 were excluded because of irrelevant studies, not related to laminitis, case reports, and no full text available, whereas 11 were duplicates from all databases. There were 39 full-text articles that remained after screening titles and abstracts. Of these articles, 32 were deemed unsuitable for this analysis because of inappropriate study designs, irrelevant parameters, and insufficient data. Ultimately, 7 studies were deemed eligible for inclusion in the meta-analysis (Figure 1).

### Characteristics of the studies included

Of the seven studies that were considered, the references used included publications from 1997 to 2019, representing 5 studies conducted in the USA, 1 study conducted in Canada, and 1 study conducted in the UK, even though certain study periods were not clearly reported. Irish Sports (n = 3), Pony (n = 5), Trotter (n = 4), Arabian (n = 10), Thoroughbred (n = 25), Quarter horse (n = 18), Morgan (n = 2), Paint (n = 5), Welsh pony (n = 2), Draft horse (n = 1), Percheron (n = 1), and another breed (n = 26) were among the horse breeds investigated. In these studies, dry needling, hemo-acupuncture, aqua-acupuncture (Aqua-AP), and electroacupuncture (EAP) were implemented. The bladder (BL)-11, BL-13, pericardium (PC)-1, heart (HT)-9, lung (LU)-1, LU-11, small intestine (SI)-9, large intestine (LI)-11, and Baihui points were reported as the primary markers for inducing acupuncture points during equine laminitis treatment. In addition, acupuncture induction was also performed at the Qian-Ti-Men, Qian-Ti-Tou, and several meridian points representing the BL, SI, LI, triple heater (TH), HT, LU, gallbladder (GB), stomach (ST), PC, conception vessels (CVs), kidney (KID), and spleen (SP). The therapy period was implemented at least twice a week for 4 consecutive weeks (Table 2) [7–13].

**Table 2:** Characteristics of the studies.

Country	Study Period	Breeds	Acupuncture method	Acupuncture point	Therapy period	References
UK	2013	Irish Sports (n = 2), Ponies (n = 2), and Trotters (n = 4)	Dry needling	(BL-10, 13, 16, 20, 22, 23, 25, 40); (LI-11, 15, 16); (SI-9); (TH-15, 16); (GB-21); and Bai-Hui	Days 1, 3, and 7	[7]
USA	2015	N/A (n = 12)	Dry needling, hemo-acupuncture, aqua-acupuncture	(ST-45); (GB-44); (BL-25, 26, 27, 67); (SI-1); (TH-1); (LI-1); (HT-9); (PC-9); (LU-11); Bai-Hui; Qian-Ti-Men; Qian-Ti-Tou	Twice weekly	[8]
USA	N/A	Arabian (n = 6)	Hemo-acupuncture	Qian Ti Men; Qian Ti Tou; Qian-Jiou	N/A	[9]
USA	N/A	Thoroughbred (n = 18), Quarter horse (n = 15), Morgan (n = 2), Paint (n = 3), Pony (n = 3), Arabian (n = 4), Welsh pony (n = 2), Draft horse (n = 1), Irish sport horse (n = 1), Percheron (n = 1)	Dry needling	(BL-10, 11, 13, 14, 15, 16, 17, 18, 19, 20, 21, 22, 23, 24, 25, 26, 27, 28, 29, 30, 35, 36, 37, 38, 39, 40, 53, 54); (CV-17); (GB-20, 21, 27); (KID-27); (LI-16, 17, 18); (LU-1); (PC-1); (SI-9, 16); (SP-11, 12, 13); (ST-7, 10, 31); (TH-15, 16); Huan-Hou; Huan-Tiao; Huan-Zhong; and Lu-Gu	N/A	[10]
USA	2015	N/A (n = 14)	Dry needling, hemo-acupuncture, aqua-acupuncture	(ST-45); (GB-44); (BL-25, 26, 27, 67); (SI-1); (TH-1); (LI-1); (HT-9); (PC-9); (LU-11); Bai-Hui; Qian-Ti-Men; Qian-Ti-Tou	Twice weekly	[11]
Canada	N/A	Paints (n = 4), quarters (n = 3), Thoroughbred cross (n = 1), Paint cross (n = 1)	Dry needling, electroacupuncture	Bai-Hui; (BL-11, 13); (PC-1); (HT-9); (LU-1, 11); (SI-9); (LI-11)	2 times per week for 4 consecutive weeks.	[12]
USA	N/A	Thoroughbred (n = 6)	Electroacupuncture	Bai-Hui; Duan-Xue; Qiang-Feng; San Yang Luo; Qian Chan Wan; Qian-Jiu	80–120 Hz for 45 min	[13]

BL=Bladder, LI=Large intestine, SI=Small intestine, TH=Triple heater, GB=Gallbladder, ST=Stomach, HT=Heart, PC=Pericardium, LU=Lung, CV=Conception vessels, KID=Kidney, SP=Spleen. N/A=Data not available

### Lameness and recovered horses

**Figure 2:**
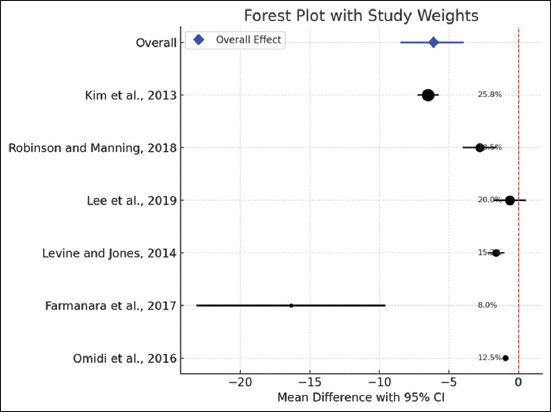
Forest plot of lameness scores across studies.

**Figure 3:**
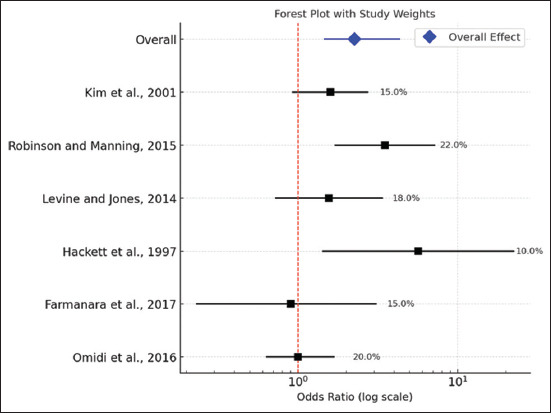
Forest plot of recovered horses across the studies.

**Figure 4:**
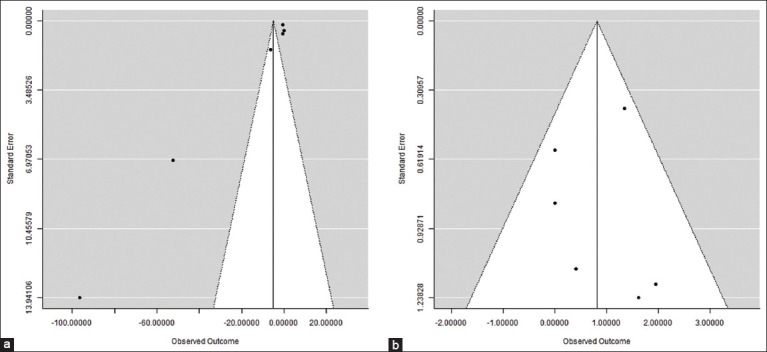
Funnel plot of (a) lameness scores and (b) recovered horses across studies.

According to the current meta-analysis, acupuncture therapy can significantly ameliorate lameness scores in horses with laminitis (mean difference = −5.008; 95% CI = −8.094–−1.923) with a high degree of heterogeneity (I^2^ = 95.81%; p < 0.001) (Figure 2). In the meantime, it was revealed that acupuncture therapy improved the laminitis-recovered horses (Odds Ratio = 2.254; 95% CI = 1.167–4.355), despite the low heterogeneity value (I^2^ = 19.39%; p = 0.287) (Figure 3). While the recovered horses displayed symmetric findings, the lameness score parameters were presented to be biasedly distributed based on the Funnel plot (Figure 4).

## DISCUSSION

The term “laminitis” can refer to various debilitating conditions resulting in functional and morphological changes within the claw. On the other hand, in horses, the term “founder” typically refers to a chronic (long-term) condition characterized by pivoting of the third phalanx (coffin bone), which is the fatal consequence of laminitis, whereas the term “acute laminitis” refers to symptoms associated with an abrupt initial attack, such as pain, discomfort, and inflammation of the dermal lamellae. The horny wall, through its vertical keratophyllin laminae, fuses with the podophyllin laminae of the keratogenous layer to form the suspensory apparatus of the third phalanx along with the epidermal lamellae of the inner hoof wall, with which they interlock [17].

Acute laminitis is characterized by hypertension of the digital and collateral arterioles, a marked increase in the local temperature of the hoof wall, and intense pain in the hooves during hammer testing. Animals also show signs of tachycardia, hyperpnea, and even toxic shock. Long-term instances typically result from one or more acute assaults [18]. When a horse has chronic laminitis, it may rest on the back of the affected limb’s hoof, which can deform the hoof and elevate the heel while also causing the front portion of the hoof wall to occasionally elongate and deform noticeably [19].

This meta-analysis revealed that earlier studies have demonstrated the effectiveness of acupuncture in reducing lameness scores and increasing the number of recovered horses. There are several methods for activating acupuncture points. One of the original forms of acupuncture is the dry needle acupuncture (DNAP) technique. It is referred to as Bai-Zhen or the “White Needle” in Traditional Chinese Veterinary Medicine (TCVM) with no intentional bleeding. In both human and veterinary therapy, acupuncture is the most widely used treatment. Depending on the species and position of the acupoints, the procedure entails inserting tiny, sterilized needles of a specific gauge and length [20]. The use of EAP as a supplement to DNAP therapies is expanding. A moderate electrical current applied through the acupuncture needles after they have been inserted into the appropriate acupoints enables more consistent, persistent, and reproducible therapeutic stimulation. It is possible to modify the electrical current’s amplitude and frequency. Different frequencies have different effects on systemic neuromodulation, and the amplitude is adjusted to the patient’s tolerance of the stimulation threshold [21]. Fluids and soluble products are injected into acupuncture points during Aqua-AP. In addition, homeopathic treatments, sterile saline, vitamin B12, and local anesthetics may also be used during Aqua-AP [22].

The fundamental concept of acupuncture point selection is the identification of body locations where stimulation will modulate ongoing physiological activity and cause favorable changes in the central nervous system [23]. The primary points for addressing lameness encompass acupoints considered to have a clinical impact on disorders related to the skeleton, tendon, or muscle. To cover every single region, the first region can be covered by choosing BL-23 and BL-11; the second region can be covered by choosing GB-34 and BL-18; and the third region can be covered by choosing BL-20. BL-54, BL-67, and ST-45 were also suggested for the hindlimb, and SI-9, SI-3, and TH-1 were suggested for any forelimb lameness [24]. Similar to the majority of ailments linked to lameness, traditional acupuncture, EAP, and aquapuncture (saline or vitamin B12 injections) are frequently used in tandem. An acupuncturist can develop a recipe for complete treatment by adding nearby points and delicate diagnostic points to the aforementioned primary points [25].

Treatment requires stimulation of points connected to each joint, which represent the joint as well as the connecting myofascial planes. The suggested illustrations are PC-6 for the carpus, SI-9 for the shoulder, BL-53 for the hip, ST-36 for the stifle, BL-60 for the tarsus, and for the distal hindlimb, KID-1 or Hou-Ti-Men (the hindlimb equivalent of Qian-Ti-Men) [26]. In a different study, bilateral EAP stimulation at 2 to 5 Hz was applied to Bai-hui, BL-11, BL-13, PC-1, HT-9, LU-1, and LU-11, as well as to SI-9 and LI-11 [20]. Numerous neural system levels, including increased opioid peptide release, increased oxytocin concentrations, and activated serotonin receptors, have been implicated in the prevention and modification of pain perception by acupuncture [27]. Moreover, the nitric oxide synthase activity near the meridians and acupoints may be elevated by acupuncture [28]. Lamellar necrosis, ischemia, and hypoperfusion are linked to laminitis [29]. According to another study, the activation and deactivation of matrix metalloproteinases (MMPs), such as MMP-2 and MMP-9, as well as a disintegrin and metalloproteinase with thrombospondin motifs-4, are linked to the destruction of the lamellar basement membrane and are important for reducing laminitis [30]. The fact that paralysis is subjective may account for the varying outcomes of acupuncture for lameness. It was discovered that acupuncture treatment decreases variations in hip elevation across all evaluation scenarios [31].

This study’s limitations include variations in parameters assessed in any investigation and linearity of findings that allow for integrated comparisons. In a single investigation, horses with laminitis had their serum cortisol, beta-endorphin, stress, and other chemical levels measured. However, most studies have focused mainly on lameness scores, qualitative gait observations, and horse recovery. We believe that several additional factors influence the healing outcomes of laminitis in horses, including feed, acupuncture points, intensity of acupuncture therapy, and cryotherapy. However, to validate the inconsistent recovery outcomes from laminitis in horses, the included studies were also compared with control groups that had the same factors in the respective studies. In general, the data from earlier studies suggesting that acupuncture therapy alters equines’ gait – treated horses move more symmetrically, indicating reduced levels of discomfort – is integrated into this meta-analysis study.

## CONCLUSION

This meta-analysis emphasized the medical evidence from previous studies that acupuncture can be used to treat horses with laminitis. Acupuncture procedures using dry needling techniques, hemo-acupuncture, aqua-AP, and EAP are focused on the Bai-Hui, Qian-Ti-Men, Qian-Ti-Tou points, and the meridian points of the BL, SI, LI, TH, HT, LU, GB, ST, PC, CVs, KID, and SP. Medical evidence has demonstrated that twice-weekly therapy for 4 consecutive weeks can raise a horse’s chances of recovery and ameliorate lameness scores. Although this meta-analysis study has demonstrated that acupuncture contributes to the recovery of laminitis-affected horses, further studies seem to be warranted to investigate whether acupuncture is beneficial when combined with feed supplements, non-steroidal anti-inflammatory medication, or physiological modifiers.

## AUTHORS’ CONTRIBUTIONS

MTEP: Conceptualized and constructed the study methodology. AP, MTEP, and FF: Curated and extracted the data. MTEP, AP, and STM: Analyzed the data and validated and visualized the figures and tables. MTEP, FF, and STM: Wrote the draft, revised, and submitted the manuscript. All authors have read and approved the final version of the manuscript.
